# Adenocarcinoma arising in sigmoid colon neovagina 53 years after construction

**DOI:** 10.1186/s12957-018-1372-z

**Published:** 2018-04-27

**Authors:** Kazunosuke Yamada, Dai Shida, Tomoyasu Kato, Hiroshi Yoshida, Shigetaka Yoshinaga, Yukihide Kanemitsu

**Affiliations:** 1 Department of Colorectal Surgery, National Cancer Center Hospital, 5-1-1 Tsukiji, Chuo-ku, Tokyo, 1040045 Japan; 2 Department of Gynecology, National Cancer Center Hospital, Tokyo, 1040045 Japan; 3 Department of Pathology and Clinical Laboratories, National Cancer Center Hospital, Tokyo, 1040045 Japan; 4 Department of Endoscopy, Gastrointestinal Endoscopy Division, National Cancer Center Hospital, Tokyo, 1040045 Japan

**Keywords:** Neovagina, Vaginal agenesis, Sigmoid colon neovagina, Adenocarcinoma, Colon carcinogenesis

## Abstract

**Background:**

In view of the rarity of vaginal agenesis, malignancy arising in the neovagina is extremely rare.

**Case presentation:**

Here, we report a 76-year-old female with an adenocarcinoma arising in the sigmoid colon neovagina which was constructed 53 years ago for congenital vaginal agenesis. Vaginal endoscopy to examine vaginal bleeding revealed a protruding lesion occupying three quarters of the lumen in the vicinity of anastomosis of the residual vagina and sigmoid colon. Transvaginal ultrasonography revealed the muscularis propria layer (hypoechoic fourth layer) to be interrupted. CT revealed no distant metastasis. Total pelvic exenteration was performed based on the diagnosis of neovaginal cancer at the anastomosis site. The 45-mm tumor showed well-differentiated adenocarcinoma with a mucinous adenocarcinoma component. Immunohistochemistry showed no p16-overexpressing tumor cells, suggesting the lack of human papilloma virus infection.

**Conclusions:**

Although rare, clinicians should be aware of cancer that arises in the ectopic intestine when anastomosed with other organs.

## Background

Neovaginal construction/reconstruction is indicated for cases of congenital vaginal agenesis which is an uncommon genital tract anomaly that is reported to occur in 1 per 4000–5000 newborn females [1, 2], as well as genetic sexual ambiguity, vaginal loss resulting from gynecologic cancer, or post-traumatic injury and male to female transsexualism [3, 4]. Multiple vaginal reconstructive techniques have been reported, including bowel flaps, skin grafts, peritoneal grafts, and musculofasciocutaneous flaps [5]. Among these, the sigmoid colon is used most often in neovaginal reconstruction, given its unique characteristics, such as its close proximity to the operative site, morphology, and capacity to replicate vaginal function. Moreover, its thicker mucosa is less vulnerable to trauma induced by coitus [6, 7].

Primary vaginal carcinoma is extremely rare, accounting for only about 2% of malignant neoplasms of the female genital tract [8]. Moreover, in view of the rarity of vaginal agenesis, malignancy arising in the neovagina is extremely rare. To date, only seven cases of adenocarcinoma arising in the neovagina have been reported in English publications, and of these, six cases of adenocarcinoma of the neovagina arose in the colon and one from the small intestine [9–15]. Here, we report a case of adenocarcinoma arising in the neovagina of a patient with Mayer-Rokitansky-Küster-Hauser syndrome [16], which refers to the congenital aplasia or severe hypoplasia of the structures that derive from the mullerian ducts, occurring 53 years after initial neovagina construction.

## Case presentation

**Fig. 1:**
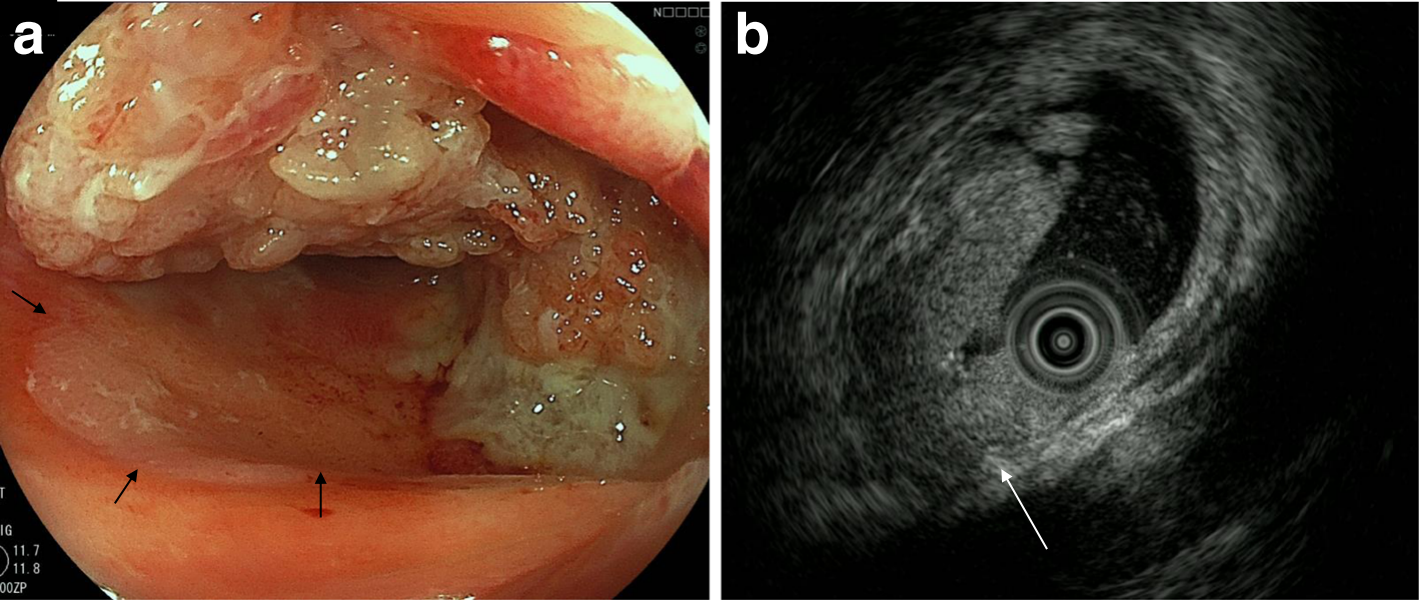
**a** Transvaginal endoscopy. Anastomosis of ectopic sigmoid colon with residual vagina (black arrows). A 45-mm protruding lesion was observed in the vicinity of the anastomosis site, occupying about three quarters of the lumen. **b** Transvaginal ultrasonography. Muscularis propria layer (hypoechoic fourth layer) was interrupted with an unclear layer pattern. Hypoechoic tumor appeared to invade beyond the colon wall (white arrow)

Fifty-three years ago, a 23-year-old Japanese woman was referred to the outpatient clinic of our hospital with primary amenorrhea. Examination revealed normal external genitalia and an absent vagina. She was diagnosed with Mayer-Rokitansky-Küster-Hauser syndrome. Hereditary disease was excluded based on family history. She underwent neovaginal construction surgery using a segment of the sigmoid colon. At the age of 45, she underwent hysterectomy for uterine myoma. At the age of 76, she visited the original outpatient clinic with the chief complaint of vaginal bleeding. Vaginal endoscopy confirmed anastomosis of the residual vagina with the sigmoid colon and revealed a 4.5-cm protruding lesion in the vicinity of anastomosis, occupying about three quarters of the lumen (Fig. 1a). Biopsy from the protruding lesion revealed well-differentiated adenocarcinoma.

**Fig. 2:**
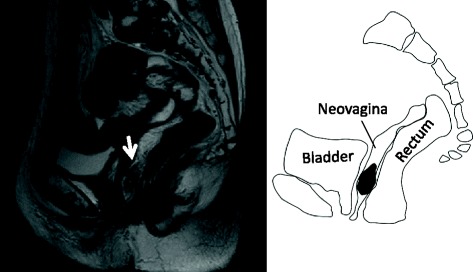
T2-weighted MR image. The neovagina existed between the bladder and rectum and extended from the vaginal opening to abdominal cavity. A 4.5-cm protruding lesion (white arrow) was detected along the posterior neovaginal wall

Transvaginal ultrasonography revealed the muscularis propria layer (hypoechoic fourth layer) to be interrupted with an unclear layer pattern and invasion of a hyperechoic tumor beyond the colon wall (Fig. 1b). The lesion was diagnosed as clinical T3 or deeper, and endoscopic resection was not indicated. T2-weighted MR imaging showed the neovagina, which extended from the vaginal opening to abdominal cavity, to exist between the bladder and rectum (Fig. 2). A 4.5-cm protruding lesion was detected along the posterior neovaginal wall. The rectum and urethra appeared to be free of invasion. CT showed no evidence of lymph node or distant metastasis. Colonoscopy of the remnant colon and rectum did not reveal any abnormalities. Tumor marker levels were as follows: CEA 2.5 ng/ml, CA19-9 20 U/ml, CA125 14 U/ml, and SCC 0.8 ng/ml, all of which were within the normal range. Based on the preoperative diagnosis of adenocarcinoma arising in the sigmoid colon neovagina 53 years after construction, we performed total pelvic exenteration for curative resection.

**Fig. 3:**
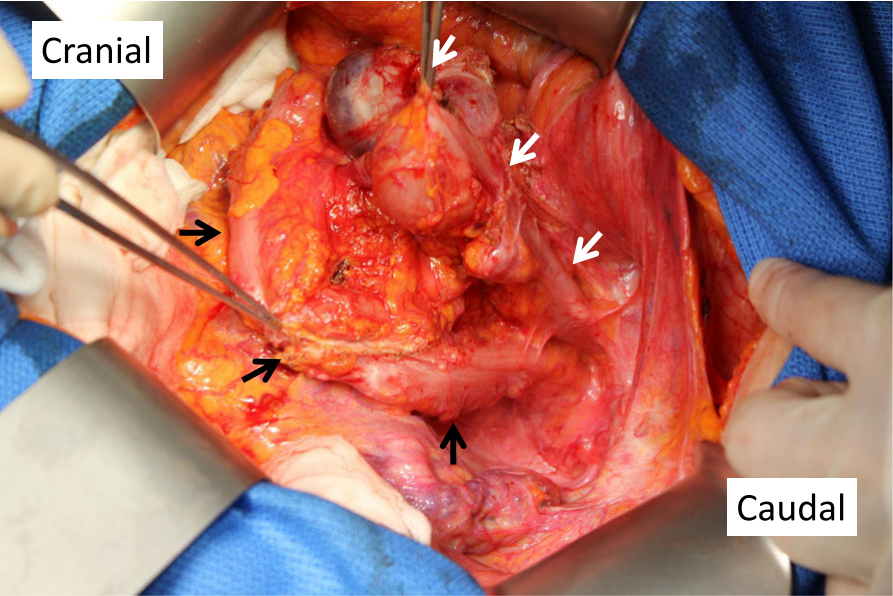
Intraoperative findings. Blind end of the neovagina was confirmed on the retroperitoneum side of the remnant sigmoid colon. In the pelvis, the neovagina passes through the antimesenteric side and anterior wall of the rectum and was anastomosed with the vaginal opening (white arrows: neovagina, black arrows: remnant sigmoid colon)

**Fig. 4:**
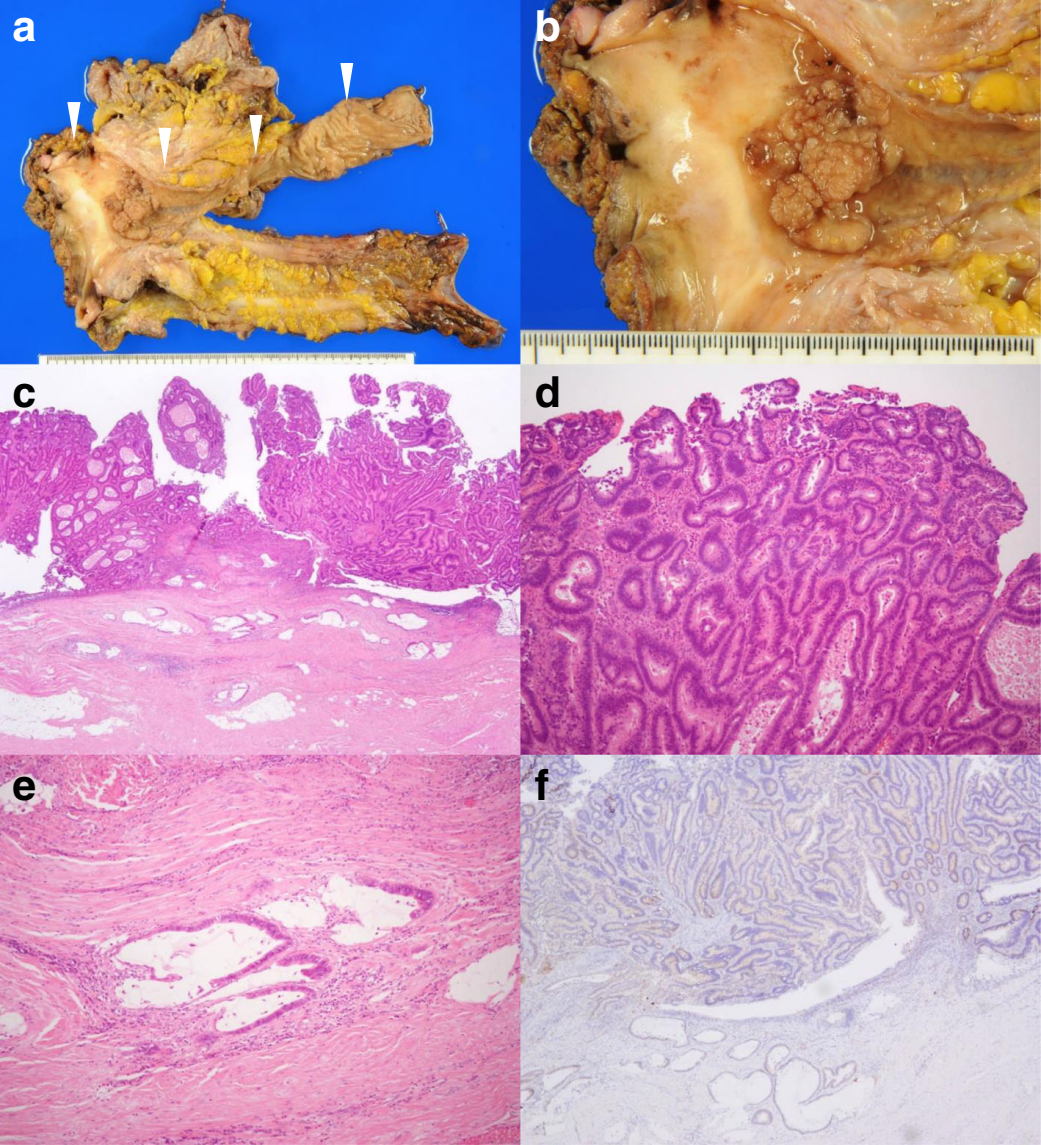
Pathological examination of resected specimen. **a** Tumor located in the neovagina constructed with ectopic sigmoid colon (white arrow heads). **b** A tan-colored papillary elevated tumor was observed in the neovagina. **c** Low magnification of the tumor (H&E, original magnification × 20). **d** At higher magnification, a well-differentiated tubular adenocarcinoma was observed in the surface area of the tumor (H&E, original magnification × 100). **e** Mucinous adenocarcinoma component in the invasive portion (H&E, original magnification × 100). **f** Immunohistochemistry revealed no p16-overexpressing tumor cells (original magnification × 40)

Intraoperatively, the blind end of the neovagina was confirmed on the retroperitoneum side of the remnant sigmoid colon. In the pelvis, the neovagina passed through the antimesenteric side and anterior wall of the rectum and was anastomosed with the vaginal opening (Fig. 3). Intra-abdominal dissemination and ascites were not found. The operative time was 8 h and 20 min, and blood loss was 385 ml. The tumor was located in the vicinity of the anastomosis site and was macroscopically a 4.5-cm protruding lesion. Adjacent tissues such as the urethra and labia minora were free of invasion (Fig. 4a, b). Pathological examination revealed that radial margins were all negative and an adequate surgical margin was obtained. Sixteen lymph nodes retrieved by lymph node dissection were all free of tumor cells. The histology showed well-differentiated tubular adenocarcinoma in the surface area of the tumor (Fig. 4d), with a mucinous adenocarcinoma component in the invasive portion (Fig. 4e). Immunohistochemistry revealed no p16-overexpressing tumor cells, suggesting the lack of human papilloma virus (HPV) infection (Fig. 4f). The carcinoma penetrated into the submucosal layer of the neovagina without any evidence of vascular or lymphatic invasion. According to the TNM classification eighth edition [17], the ectopic colon cancer was categorized as T2N0M0, or stage I colon cancer, and thus, the patient did not undergo adjuvant therapy. The postoperative course was uneventful, and she was discharged on postoperative day 29. At present, 7 months postoperatively, the patient has neither symptoms nor apparent signs of recurrence.

## Discussion and conclusions

Neovaginal carcinoma is extremely rare, whereas vaginal cancer which accounts for about 2% of all gynecological malignancies [8]. Schober et al. summarized the 30 cases of neovaginal carcinoma reported worldwide; of these, eight were adenocarcinomas arising from the intestinal grafts, and 17 were squamous cell carcinoma arising from the skin and the skin graft [18]. Thus, the pathological characteristics of this cancer are likely associated with the tissue used for reconstruction.

A literature search of “neovagina” and “adenocarcinoma” using PubMed yielded only seven cases of neovaginal adenocarcinoma (in which the intestine was used for construction) in the English literature. Of these, six cases of neovaginal adenocarcinoma originated from the colon and one case from the small intestine [9–15]. Our case is the seventh case of neovaginal adenocarcinoma involving the colon. Operative methods differed by case. For example, Hiroi et al. [11] performed total resection of the neovagina and bilateral salpingo-oophorectomy. Bogliolo et al. [13] performed subtotal vaginectomy and total radical hysterectomy with left salpingo-ovariectomy. Kita et al. [14] initially planned to remove only the neovagina, but switched to posterior pelvic exenteration due to substantial adhesion of the neovagina to the rectum and bladder. Thus, adhesion is an important factor to consider when planning surgery. As discussed above, there is no consensus operative strategy for these patients. In the present case, we diagnosed the patient with advanced cancer according to transvaginal ultrasonography and also anticipated strong adhesion of the neovagina to the rectum and bladder. Based on this, we considered it difficult to resect only the neovagina. In the end, we performed total pelvic exenteration in order to achieve complete resection with microscopic negative margins (R0).

Numerous risk factors associated with colon cancer have been reported, including age, sex, race, dietary habits, consumption of red meat, obesity, toxic substances such as tobacco, alcohol intake, and host factors [19–22]. Chronic inflammatory processes in the colon including ulcerative colitis and Crohn’s disease are also associated with an increased risk of developing colorectal cancer [23]. The present case report clearly demonstrated the potential occurrence of malignancy in ectopic intestinal tissue used for neovaginal construction. It is easily conceivable that the ectopic colon, although anatomically excluded and separate from the influence of two potential factors that promote cancer development (i.e., bile acid and direct contact with potentially carcinogenic food), retains the basic properties of the remaining large bowel, including genetic factors (e.g., oncogenic activation) involved in cancer development. Notably, as with our present case, neovaginal adenocarcinoma (which involved the sigmoid colon) was located in the vicinity of the anastomosis site in four of five previously reported cases. Other than the neovagina, secondary malignancy at the uroenteric anastomosis site is a well-documented long-term complication after ureterosigmoidostomy. Ectopic colon cancer occurred at the ureterocolonic junction with an 8.5- to 10.5-fold increased risk of colon carcinoma compared to the general population [24]. Moreover, p53 mutations, which are indicative of genetic instability, are known to occur at the ileovesical anastomosis site in patients who have undergone clam ileocystoplasty, which results in neoplasia after ureterosigmoidostomy at uroenteric anastomosis sites [25]. Chemical stimulants in semen and urine may have contributed to malignancy in these previous cases. These reports, as well as our present case, suggest that intestinal anastomosis with other organs may increase the risk of cancer.

HPV infection is a well-established cause of invasive cervical cancer, and there is an increasing body of evidence strongly linking HPV DNA with other anogenital cancers (anus, vulva, vagina, and penis) and head and neck cancers (particularly the oropharynx, base of the tongue, and tonsils) [26]. However, with respect to adenocarcinoma occurring in the sigmoid colon neovagina, our case was immunohistochemically negative for the p16 protein, as were the three previous cases for which p16 was examined. These results suggest that adenocarcinoma occurring in the sigmoid colon neovagina may be independent of HPV infection.

In conclusion, we encountered a patient with a rare carcinoma in the sigmoid colon neovagina. To achieve complete resection with microscopic negative margins for advanced neovaginal cancer, total pelvic exenteration is a good surgical option. Although rare, clinicians should be aware of cancer arising in the ectopic intestine when anastomosed with other organs, given the persistent risk of malignant transformation.

## Abbreviations

HPV: Human papilloma virus
